# Effect of Periodic Vehicle Inspection on Road Crashes and Injuries: A Systematic Review

**DOI:** 10.3390/ijerph18126476

**Published:** 2021-06-15

**Authors:** Luis Miguel Martín-delosReyes, Pablo Lardelli-Claret, Laura García-Cuerva, Mario Rivera-Izquierdo, Eladio Jiménez-Mejías, Virginia Martínez-Ruiz

**Affiliations:** 1 Department of Preventive Medicine and Public Health, School of Medicine, University of Granada, Avenida de la Investigación 11, Edificio A, 8ª planta, 18016 Granada, Spain; luismiguelmr@ugr.es (L.M.M.-d.); lardelli@ugr.es (P.L.-C.); laugarcia92@gmail.com (L.G.-C.); mariorivera@ugr.es (M.R.-I.); eladiojimenez@ugr.es (E.J.-M.); 2 Doctorate Program in Clinical Medicine and Public Health, University of Granada, 18071 Granada, Spain; 3 Centros de Investigación Biomédica en Red de Epidemiología y Salud Pública (CIBERESP), 28029 Madrid, Spain; 4 Instituto de Investigación Biosanitaria de Granada (ibs.GRANADA), 18012 Granada, Spain; 5 Department of Teaching and Research in Family Medicine, School of Medicine, University of Granada, 18016 Granada, Spain; 6 Service of Preventive Medicine and Public Health, Hospital Universitario Clínico San Cecilio, 18016 Granada, Spain

**Keywords:** vehicle inspection, road crash, road injury, motor vehicles

## Abstract

This systematic review was conducted to determine the effect of periodic motor vehicle inspections on road crashes and injuries, compared to less exposure to periodic inspections or no inspections. The Medline, Web of Science, and Scopus databases were used to search the literature. Ecological studies were specifically excluded. A reverse search of the results with these databases and of other identified narrative reviews was also performed. Of the 5065 unique references initially extracted, only six of them met the inclusion criteria and were selected for review: one experimental study, two cohort studies with an internal comparison group, two cohort studies without a comparison group, and one case–control study. Two authors independently extracted the information and assessed the quality of each study. Due to the heterogeneity of the designs and the intervention or comparison groups used, quantitative synthesis of the results was not attempted. Except for the case–control study, which showed a significant association between road crashes and the absence of a valid vehicle inspection certificate, the other studies showed either a small reduction in crash rates (around 9%), no association, or a higher crash rate in vehicles with more inspections. In all observational studies, the risk of residual confounding bias was significant and could have explained the results. Therefore, although the research reviewed here suggests that periodic inspection may be associated with a slight reduction in road crashes, the marked heterogeneity along with probable residual confounding in most reports prevented us from establishing causality for this association.

## 1. Introduction

Vehicle defects have been identified as one of the contributing causes of road crashes. The proportion of road crashes attributed to vehicle defects estimated in previous reports varied widely from 3% to as much as 19% in developed countries [1,2], and the highest rate reported as 27% in developing countries [3]. This relationship is the main reason used to justify the implementation, in many countries, of vehicle technical inspection (VTI) programs as a legal requisite for roadworthiness, given that such inspections can detect technical defects and thereby prevent crashes [2,4,5,6].

However, novel technologies and advances in traffic safety in recent years have led to declines in road crash rates, and as a result, some countries or regions within countries have opted to abolish this legal requirement [7,8]. According to reports by the National Highway Traffic Safety Administration in the USA, the risk of road crashes associated with driving a vehicle manufactured before 2000 is 71% higher than for vehicles manufactured in 2010 and later [9]. This finding, along with similar data, constitutes the main argument supporting economic subsidies intended to lower the mean age of vehicles on the road, instead of building, maintaining, and operating specialized VTI centers in those countries with VTI policies in effect [8]. Previous studies (none of which were systematic reviews) on this issue were inconclusive regarding the usefulness of VTI in reducing road crashes. The article by Jarosinski [4] noted the problem of underestimating the effects of vehicle technical defects in the causal chain of road crashes—a consequence of the fact that most published studies were based on registries maintained by police agencies. It was noted that officers at the scene lacked sufficient resources to record information on all factors that might have caused the crash, particularly those attributable to technical defects in the vehicles involved. According to Jarosinski, this accounted for the differences between the findings of studies on the usefulness of VTI. The review by Rechnitzer et al. [2] concluded that studies published up to the time their review was written have evident methodological shortcomings that rendered them unable to evaluate the relationship between VTI and automobile defects rigorously. These authors admitted that most such studies were rather old and that the characteristics of vehicles on the road had changed substantially in the meantime, as shown by the increase in warranty periods offered by car manufacturers. 

It appears evident that maintaining mandatory VTI should be backed by evidence of its efficacy in reducing road crashes, rather than solely by theoretical arguments, based on its proven usefulness in detecting (and correcting, when necessary) vehicle defects potentially associated with a greater risk of causing road crash [2,4,5,6,7,8]. In principle, the high cost of maintaining specialized testing and inspection centers and the contradictions among classic review articles available to date would appear not to justify the implementation of systematic inspections. The present study was undertaken with the aim of systematically reviewing analytical studies (i.e., studies capable of providing evidence of causality) published to date to shed further light on the usefulness of VTI in order to quantify the effect of periodic motor vehicle inspection on the rates of road crashes and related injuries.

## 2. Materials and Methods

This systematic review was done in accordance with the guidelines in the PRISMA statement [10]. The elements used to define the research question according to the PICOS system were as follows:

- Population: Motor vehicles.
- Intervention: Periodic vehicle inspection.
- Comparison: Any vehicle status resulting in lower exposure to periodic inspection compared to the intervention group (for example, no inspection, lower frequency of inspection, or greater time elapsed since the most recent inspection).
- Outcomes: Road crashes, injuries resulting from road crashes, deaths resulting from road crashes.
- Study design: Analytical studies based on individual data, i.e., with the vehicle and/or its driver as the unit of study. Ecological studies were specifically excluded because the level of causal evidence they provide is considered weak [11,12].

Studies published in English, German, Spanish or Portuguese were included. No restrictions on the year of publication were used. The reference lists of all articles initially extracted were used for the reverse review. Although review articles were excluded from the initial searches, their Reference lists were used as sources of information to identify additional studies containing primary data. The information sources used for bibliographic searches were Medline, Web of Science (WOS), and Scopus. The search strings used for each database are shown below:

Scopus: 

TITLE-ABS-KEY(vehicle* OR motor* OR car*) AND TITLE-ABS-KEY (inspection* OR maintenance) AND TITLE-ABS-KEY (traffic* OR road*) AND TITLE-ABS-KEY (accident* OR crash* OR casualt* OR mortalit* OR death OR injur* OR safety OR roadworthiness)

WOS: 

TOPIC: (vehicle* OR motor* OR Car*) AND TOPIC: (inspection* OR maintenance) AND TOPIC: (traffic* OR road*) AND TOPIC: (Accident* OR Crash* OR Casualt* OR Mortalit* OR Death* OR Injur* OR safety OR roadworthiness)

Medline:

((((vehicle*[Title/Abstract] OR motor*[Title/Abstract] OR Car*[Title/Abstract])) AND (inspection*[Title/Abstract] OR maintenance[Title/Abstract])) AND (traffic*[Title/Abstract] OR road*[Title/Abstract])) AND (Accident*[Title/Abstract] OR Crash*[Title/Abstract] OR Casualt*[Title/Abstract] OR Mortalit*[Title/Abstract] OR Death[Title/Abstract] OR Injur* OR safety OR roadworthiness) [Title/Abstract])

Two authors were responsible for the final selection of articles for inclusion. Each author independently evaluated and extracted the relevant information from each article included for analysis. Information from each article was recorded for the following variables: bibliographic reference, type of design, study population, intervention or exposure, comparison, duration of follow-up, outcome, estimated effect size, main results and quality evaluation, risk of biases, and limitations. To evaluate the quality of observational studies, we used the Newcastle–Ottawa Scale [13], and for experimental studies, we used the Jadad scale [14]. A senior researcher resolved discrepancies between the two evaluators. As explained in the Results section, the marked heterogeneity among studies regarding their design, exposure groups, and comparison groups prevented us from undertaking a quantitative synthesis of the results from all studies (meta-analysis).

## 3. Results

**Figure 1:**
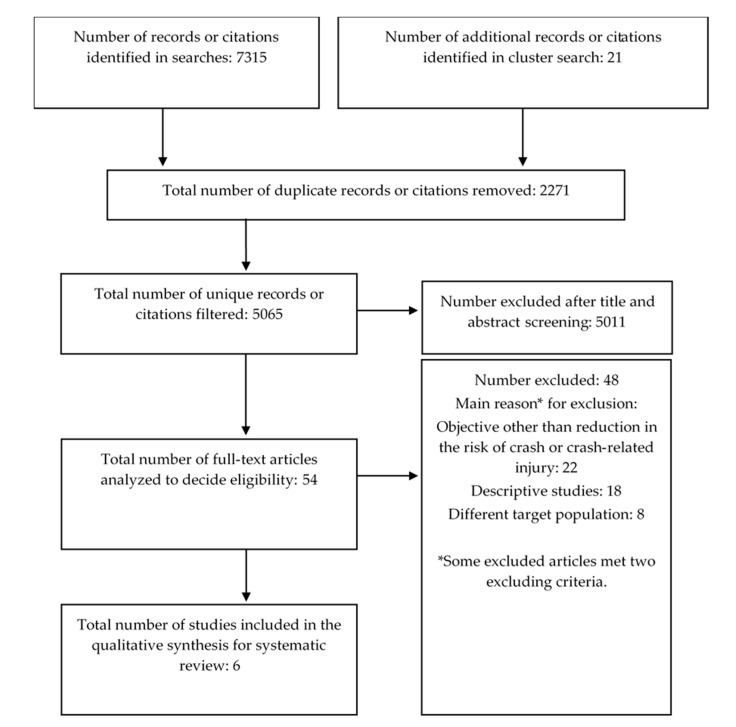
Flowchart of the article selection process.

Figure 1 shows the flow diagram for the selection of sources included in the present systematic review. The initial search yielded 5065 unique references, most of which were excluded on the basis of their title and abstract. The main reason for exclusion was a study objective different from any of the outcomes specified in our PICOS outline (for example, engineering studies that evaluated the technical quality of VTI facilities). Reading the full text of all 54 articles remaining after this initial screen showed that only six fulfilled all inclusion criteria defined in our PICOS strategy. The reasons for exclusion for the other 48 articles are shown in Figure 1.

Table 1 summarizes the main characteristics of each of the six articles included for systematic review, their overall quality, and potential biases. The main results from each study in chronological order are summarized below.

- Schroer and Peiton [15] found a significant 9.1% reduction in crash rate in vehicles between 5 and 10 years old that underwent VTI compared to uninspected vehicles. The authors noted that their comparison might have been distorted by roadside inspections in the state (Alabama, USA) where the study was done. In addition, exposure to VTI was voluntary, a factor that seriously compromised the comparability of their exposed and unexposed groups.
- White [16] evaluated the effect of the number of weeks elapsed since the most recent inspection on crash rates and observed a statistically significant positive correlation. Although the author adjusted the estimate for the expected change in rates in the absence of VTI exposure, and aside from the quality of the data used in this analysis, the results may have been biased by the effect of other time-related variables in vehicles or drivers that were not controlled for, such as intensity of exposure.
- Fosser et al. [17] published the only experimental study carried out to date and randomly assigned vehicles to one of three comparison groups (no inspection, a single inspection, annual inspections). No significant differences were seen in the number of road crashes per 1000 vehicle days. However, the frequent random roadside inspections in Norway during the study period (which involved up to 20% of all vehicles yearly) might have diluted the possible beneficial effect of VTI exposure. It should be noted that this study was an open trial, dropouts from the scheduled interventions were reported, and the vehicles included were limited to those between 7 and 11 years old.
- Blows et al. [18] used a case–control design to compare drivers involved in road crashes with victims vs. a random sample of drivers on the road. In both groups, telephone interviews were used to record whether the vehicle had a VTI certificate. A significant association was found between not having a certificate of inspection and being involved in a crash, with an odds ratio ranging from 1.87 to 3.08 depending on how missing values were handled for the exposure variable. Although the estimate was adjusted for several confounding factors, the type of design and method used to record exposure (self-report) raised questions regarding the causal nature of the association found.
- Christensen and Elvik [19] used a pre–post design to compare the outcome incidence (crash rate) in a single group of vehicles studied before and after exposure to one, two, or three inspections. Although inspection was associated with a reduction in vehicle defects, and the presence of defects was associated with higher crash rates, the analysis unexpectedly showed that crash rates increased after inspection. The authors suggested this finding might be attributable to a risk compensation phenomenon, i.e., after inspection, drivers may have believed their vehicle to be safer and consequently engaged in more risky behaviors. It should be noted that the causal evidence from pre–post studies is weak. Moreover, the reliability of the outcome evaluation in this study was questionable given that it was dependent on whether accident claims were filed with the drivers’ insurance company. The rate of reporting may differ depending on the severity of the crash, resulting in distortions in the relationship between inspections and accident rates. A further limitation was the lack of control for confounders related to driver characteristics, which were not recorded in this study.
- Keall and Newstead [20] compared crash rates in vehicles that underwent annual inspection vs. vehicles inspected biannually and found a slight but significant 8% reduction in the crash rate in the latter group after adjusting for differences in vehicle age in the two groups (vehicles inspected annually had a mean age less than 7 years whereas vehicles inspected every six months were 7 years old or older).

## 4. Discussion

The present systematic review raises several important issues. The first to consider is the low number of studies that used methods that make it possible to evaluate the causality of the association between exposure to periodic inspections and road crashes and/or injuries resulting from crashes. Many of the articles reviewed here, especially among older ones, reported ecological studies in which the unit of study was not vehicles or drivers but administrative bodies (states, counties, etc.). These studies are based on comparing crash rates among entities with different levels of implementation of VTI programs [7,8,21,22,23,24]. As noted above, the causal evidence these studies provided was weak given that they were potentially affected by the well-known issue of ecological fallacy [11,12].

Although it is possible to obtain a common effect estimate (the percent variation in risk of the outcome) in all studies except one, there are many sources of heterogeneity across studies that, taken altogether, made it inadvisable to attempt a meta-analysis. Regarding the comparisons assessed, two studies used a dichotomous comparison (inspection vs. no inspection), whereas three investigated—in addition to the effect of non-inspection—various categories of frequency of exposure. One study included no nonexposed vehicles but compared two frequencies of inspection, i.e., annual and biannual, whereas in another study, the exposure variable was based on the time elapsed since the most recent inspection. On the other hand, the study designs were also markedly heterogeneous: one experimental study used random allocation, two were observational cohort studies, one was a case–control study and two were follow-up studies that lacked a concurrent control group. Third, although all but one study chose road crashes as the outcome, the source of information was different depending on the study: while three studies used state administrative registries (which tend to underreport less severe crashes and usually exclude those with only material damages), the other two used information on accidents provided by private insurance companies, which covers all types of crashes self-reported by policyholders. Finally, the selected publications covered a long period: from 1978 to 2013. A high degree of heterogeneity across studies is therefore expected due to time-related changes in the types of vehicles (progressively incorporating new protective and driver-assistant devices) and the type of compulsory inspections required by changes in legislation.

Two characteristics shared by most of the six studies were the sources of information and the outcomes investigated. One commonality among all studies except the case–control one by Blows et al. [18] was the use of data from secondary sources (i.e., traffic registries not originally intended for research purposes, such as public or insurance company databases to obtain information on registered vehicles, inspected vehicles, and road crashes). Although this procedure obviates the influence of potential differential information biases on outcome evaluations (given that the crash registries are independent of exposure to or the frequency of inspection), it has the drawbacks inherent in the use of secondary sources of information: (1) crash registries are likely to be incomplete to some degree, (2) the samples of vehicles used for analysis may not be representative of the entire population of vehicles on the road, (3) the information in databases may not be entirely reliable, and (4) information is lacking in other variables (i.e., potential confounders) that may affect the associations. This last pitfall is especially relevant in observational studies because of the likelihood that the associations observed between VTI and crash rates will be confounded by other factors related to driver and vehicle characteristics and the intensity and type of vehicle exposure to traffic. 

A second characteristic common to all studies reviewed here except the case–control one is the outcome used for comparison, i.e., road crash rates in subgroups of vehicles defined according to their exposure status. In the case–control study, on the other hand, the case group consisted of drivers of vehicles involved in road crashes with fatalities or injuries requiring hospitalization. 

As noted above, the study by Blows et al. [18] differed substantially from the other five studies reviewed here in design (case–control), type of exposure recorded (driver self-reports of having an inspection certificate), type of outcome (crashes with fatalities or injuries requiring hospitalization), and information sources (active search for cases, random sampling of controls, and telephone interviews in both groups). It is thus unsurprising that their results differed most clearly from those of the other five studies. Blows and colleagues reported a strong risk association between lack of an inspection certificate and the odds of death or injury from a road crash, whereas the remaining studies found either no association between vehicle inspection and crash rates [17,19] or a weak association [15,16,20]. 

A priori, and in light of the estimated contribution of vehicle-dependent factors on the risk of involvement in a road crash [2,4,5,6], the strong protective association (odds ratios between 1.46 and 4.86) between inspection (which would serve, theoretically, to detect and correct vehicle failures or defects) and the crash rate found in this case–control study seems implausible. In this connection, estimates such as those reported in the studies by Keall and Newstead [20] or Schroer and Peyton [15], who found risk reductions of approximately 9%, seem much more realistic. However, attempts to determine whether this association is causal or not face two obstacles:

- Given the low strength of association and the fact that it was estimated from observational studies, it could be attributable to residual confounding (i.e., the association appears due to the effects of one or more non-measured variables related to VTI exposure which are the true causes of the decreased risk of the crash of inspected vehicles compared to non-inspected ones). As discussed above, a common problem with studies of this type is the difficulty of controlling for confounders when secondary information sources are used.
- Although periodic inspections may provide other benefits apart from their impact on crash rates (mainly by reducing environmental pollution) [4,25,26] the cost-benefit ratio of strategies to prevent road crashes based on maintaining periodic vehicle inspection programs or increasing the frequency of inspections may be low, as some authors have discussed previously [7,8].

Several potential limitations in our review merit consideration. First, publication bias cannot be ruled out: we were unable to access some grey literature and are aware that the results in these sources may differ systematically from those in the articles included in this review. Second, the resources available did not allow us to include articles published in Chinese [26,27,28]. Third, the aforementioned heterogeneity depending on the time which had elapsed between the studies should also be noted, raising the question of comparability between periodic inspections carried out in different decades. 

## 5. Conclusions

The main conclusions that can be drawn from the present systematic review can be summarized as follows:

- Despite the extended time period used to search for relevant publications, very few studies met our minimum methodological requirements for providing causal evidence for the possible effect of periodic vehicle inspection on road crash rates.
- Heterogeneity across studies was considerable regarding the initial hypothesis and design characteristics, and this precluded any attempt to achieve a quantitative synthesis of their results. Despite this obstacle, from a qualitative perspective, the general pattern of findings suggests that periodic inspection is associated with a slight reduction in road crashes.
- In overall terms, the studies included in this review were compromised by a variety of methodological limitations, most related to their observational design and the limited information available. Therefore, the causal contribution of VTI programs to the reduction in road crash rates could not be definitely confirmed.

## Figures and Tables

**Table 1:** Main characteristics of the selected studies.

- Author - (Year of Publication) - Study Area	- Study Design - Study Period - Length of Follow-Up - Data Sources	- Study Populations - Intervention and Comparison	- Outcome - Measure of Effect	Main Result (*p* Value or 95% Confidence Interval)	Quality Assessment Sources of Bias
Schroer, B. J. & Peiton, W. P. (1978) Huntsville (Alabama, USA)	Retrospective cohort study April 1975 to December 1976 21 months Linkage of pre-existing databases: - Vehicles registered - Vehicle inspected - Road crashes	8494 inspected vehicles 30,089 non-inspected vehicles	Road crash rates (per vehicles) Percent change	9.1% reduction in inspected vehicles (one-tailed test: *p* < 0.05)	NOS ^1^: 7 Selection: 4 Comparability: 1 Outcome: 2 Secondary sources of information Voluntary assignment of exposure High risk of confounding
White, W. T. (1985) Levin township (New Zealand)	Retrospective cohort study 1980–1984 26 weeks Linkage of pre-existing databases: - Vehicles inspected - Road crashes	9714 cars inspected; 5898 cars inspected at least twice	Road crash rates (per inspected cars) Coefficient of the variable “weeks after the last inspection” in a Poisson regression model	0.028 (one-tailed test: *p* = 0.0002)	NOS ^1^: 6 Selection: 3 Comparability: 1 Outcome: 2 Secondary sources of information High risk of confounding
Fosser, S. (1992) Norway	Randomized controlled trial 1986–1990 Three 1-year periods Primary data about inspections Data accidents (insurance companies)	204,000 vehicles, including vans and passenger cars Three comparison groups: (1) 4600 cars inspected annually between 1986 and 1988 (2) 4600 vehicles inspected once in 1986 (3) 112,000 non- inspected cars (Control group)	Road crash rates (per car-days) Not estimated	No statistically significant differences between rates in the three groups	Jadad scale ^2^: 2 Dropouts from scheduled interventions No blinding
Blows, S. et al. (2003) Region of Auckland (New Zealand)	Prospective case–control study March 1998 to July 1999 Face-to-face or telephone interviews	Cases: 571 hospitalized drivers of passenger cars, vans, and light industrial vehicles Controls: 588 drivers of non-accident vehicles, obtained by random cluster sampling	Frequency of being in possession of a Warrant of Fitness or vehicle inspection certificate Odds ratio	2.67 (1.46, 4.86)	NOS ^1^: 6 Selection: 4 Comparability: 2 Outcome: 0 Type of design (case–control) Information (recall) bias Residual confounding
Christensen, P. &Elvik, R. (2007) Norway	Retrospective pre–post cohort study 1998–2005 Up to 5 years Linkage of pre-existing databases: -Inspections (public road administration) - Crashes (insurance company)	253,098 passenger cars observed before and after one, two, or three inspections	Road crash rates (per car) Percent change	Vehicles with one inspection: +2.6% (−0.7%, 6.0%) Vehicles with two inspections: +8.4% (3.9%, 13.2%) Vehicles with three inspections: +4.0% (−23.6%, 41.5%)	NOS ^1^: 5 Selection: 3 Comparability: 0 Outcome: 2 Type of design (pre–post) Secondary sources of information High risk of confounding
Keall, M.D. & Newstead, S. (2013) New Zealand	Retrospective cohort study 2003–2009 Up to 6 years Linkage of pre-existing databases: - Crash data - Licensing data - Inspection data	2,710,797 vehicle-years Compare vehicles subject to annual inspections (aged 6 years or less) versus vehicles subject to 6-month inspections (aged 7 years or more)	Road crash rates (per vehicle-year) Percent change	8% reduction in vehicles inspected every 6 months (0.4%, 15%)	NOS ^1^: 6 Selection: 3 Comparability: 1 Outcome: 2 Secondary sources of information Risk of confounding

^1^ Newcastle-Ottawa scale for analytic observational studies [13]. ^2^ Jadad scale for experimental studies [14].

## Data Availability

Not applicable.

## References

[1] Cuerden R.W., Edwards M.J., Pittman M.B. (2011). Effect of Vehicle Defects in Road Accidents.

[2] Rechnitzer G., Haworth N., Kowadlo N. The Effect of Vehicle Roadworthiness on Crash Incidence and Severity (No. 164). https://www.monash.edu/muarc/archive/our-publications/reports/muarc164.

[3] Taneerananon P., Chanwannnakul T., Suanpaga V., Khompratya T., Kronprasert N., Tanaboriboon Y. (2005). An Evaluation of the Effectiveness of Private Vehicle Inspection Process in Thailand. J. East Asia Soc. Transp. Stud..

[4] Jarosiński W. (2014). Periodic Technical Inspections of Vehicles and Road Traffic Safety with the Number of Road Accidents Involving Fatalities. Eksploat. Niezawodn. Maint. Reliab..

[5] Cairns S., Rahman S., Anable J., Chatterton T., Wilson R.E. (2017). Vehicle Inspections—From Safety Device to Climate Change Tool.

[6] Petit L. (2014). El factor humano en el sistema tránsito y seguridad vial y el modelo interaccional comportamental de tránsito. PSIENCIA Rev. Latinoam. Cienc. Psicológica.

[7] Hoagland A., Woolley T. (2018). It’s No Accident: Evaluating the Effectiveness of Vehicle Safety Inspections. Contemp. Econ. Policy.

[8] Das S., Geedipally S.R., Dixon K., Sun X., Ma C. (2019). Measuring the Effectiveness of Vehicle Inspection Regulations in Different States of the U.S. Transp. Res. Rec..

[9] National Highway Traffic Safety Administration (2013). How Vehicle Age and Model Year Relate to Driver Injury Severity in Fatal Crashes.

[10] Hutton B., Catalá-López F., Moher D. (2016). The PRISMA Statement Extension for Systematic Reviews Incorporating Network Meta-Analysis: PRISMA-NMA. Med. Clin..

[11] Zeoli A.M., Paruk J.K., Pizarro J.M., Goldstick J. (2019). Ecological Research for Studies of Violence: A Methodological Guide. J. Interpers. Violence.

[12] Herger N. (2020). On the Ecological Fallacy in Discrete-Choice Models. J. Choice Model..

[13] Wells G.A., Shea B., O’Connell D., Peterson J., Welch V., Losos M., Tugwell P. The Newcastle-Ottawa Scale (NOS) for Assessing the Quality of Nonrandomised Studies in Meta-Analyses. http://www.ohri.ca/programs/clinical_epidemiology/oxford.asp.

[14] Jadad A.R., Moore R.A., Carroll D., Jenkinson C., Reynolds D.J., Gavaghan D.J., McQuay H.J. (1996). Assessing the quality of reports of randomized clinical trials: Is blinding necessary?. Control. Clin. Trials.

[15] Schroer B.J., Peyton W.F. (1979). The Effects of Automobile Inspections on Accident Rates. Accid. Anal. Prev..

[16] White W.T. (1986). Does Periodic Vehicle Inspection Prevent Accidents?. Accid. Anal. Prev..

[17] Fosser S. (1992). An Experimental Evaluation of the Effects of Periodic Motor Vehicle Inspection on Accident Rates. Accid. Anal. Prev..

[18] Blows S., Ivers R.Q., Connor J., Ameratunga S., Norton R. (2003). Does Periodic Vehicle Inspection Reduce Car Crash Injury? Evidence from the Auckland Car Crash Injury Study. Aust. N. Z. J. Public Health.

[19] Christensen P., Elvik R. (2007). Effects on Accidents of Periodic Motor Vehicle Inspection in Norway. Accid. Anal. Prev..

[20] Keall M.D., Newstead S. (2013). An Evaluation of Costs and Benefits of a Vehicle Periodic Inspection Scheme with Six-Monthly Inspections Compared to Annual Inspections. Accid. Anal. Prev..

[21] Buxbaum R.C., Colton T. (1966). Relationship of Motor Vehicle Inspection to Accident Mortality. JAMA.

[22] Fuchs V.R., Leveson I. (1967). Motor Accident Mortality and Compulsory Inspection of Vehicles. JAMA.

[23] Colton T., Buxbaum R.C. (1968). Motor vehicle inspection and motor vehicle accident mortality. Am. J. Public Health Nations Health.

[24] Fridstrøm L., Ingebrigtsen S. (1991). An aggregate accident model based on pooled, regional time-series data∗. Accid. Anal. Prev..

[25] Schifter I., Díaz L., Vera M., Guzmán E., Durán J., Ramos F., López-Salinas E. (2003). Evaluation of the Vehicle Inspection/Maintenance Program in the Metropolitan Area of Mexico City. Environ. Sci. Technol..

[26] Wu Y., Zhang S., Hao J., Liu H., Wu X., Hu J., Walsh M.P., Wallington T.J., Zhang K.M., Stevanovic S. (2017). On-Road Vehicle Emissions and Their Control in China: A Review and Outlook. Sci. Total Environ..

[27] Fang H., Du Z.C. (2009). Development of Remote Monitoring System of Vehicle Inspection Lane. Comput. Technol. Dev..

[28] Liu Y.H., Ma Z.P., Xia J.Z., Wang M. (2011). Research on Control System of Vehicle Inspection Line Based on Fieldbus. China Meas. Test.

